# Turned stem tension band technique in reverse total shoulder arthroplasty for proximal humeral fracture can achieve high tuberosity healing rates regardless of the vertical sutures

**DOI:** 10.1016/j.jseint.2024.08.186

**Published:** 2024-08-30

**Authors:** Kazumasa Takayama, Hiromu Ito

**Affiliations:** Department of Orthopaedics, Kurashiki Central Hospital, Kurashiki, Okayama, Japan

**Keywords:** Turned stem tension band technique, Proximal humeral fracture, Tuberosity, Tension band, Reverse shoulder, Bone resorption, Tomosynthesis, Reduction loss

## Abstract

**Background:**

The importance of tuberosity healing in reverse total shoulder arthroplasty for proximal humeral fractures (PHFs) has been recognized. The turned stem tension band (TSTB) technique has been applied to tuberosity repair, and high bone healing and low reduction loss rates have been reported. Vertical sutures were added to the original method to reinforce fixation. We hypothesized that vertical sutures would be unnecessary in case the supraspinatus tendon was resected. This study aimed to compare the TSTB technique with or without vertical suturing for PHFs and evaluate the rates of tuberosity healing and reduction loss.

**Methods:**

Thirty five patients (vertical suture group: 18 cases and nonvertical suture group: 17 cases) underwent reverse total shoulder arthroplasty for complex PHFs using the TSTB technique. We evaluated the postoperative range of motion, the American Shoulder and Elbow Surgeons score, tuberosity healing rates, and reduction loss.

**Results:**

The vertical and nonvertical suture groups showed no significant difference in flexion (119 ± 33° vs. 124 ± 23°, *P* = .95), abduction (116 ± 35° vs. 115 ± 27°, *P* = .78), external rotation (27 ± 12° vs. 21 ± 8°, *P* = .16), internal rotation (6 ± 4° lumbar 3 level vs. 6 ± 4°, lumbar 3 level *P* = .87), the American Shoulder and Elbow Surgeons (77.3 ± 10.7 vs. 81.6 ± 6.3, *P* = .59), Numerical Rating Scale scores (1.2 ± 0.9 vs. 0.8 ± 0.9, *P* = .13), and tuberosity reduction loss (*P* = .34). The tuberosity healing rate in both groups was 100%.

**Conclusion:**

The TSTB technique for PHFs provided high tuberosity healing and low reduction loss rates regardless of vertical sutures.

Proximal humeral fractures (PHFs) are common in older adults. Most fractures have been successfully treated nonoperatively^3^; however, internal fixation should be considered in displaced fractures.^3^^,^^35^^,^^44^ Hemiarthroplasty and reverse total shoulder arthroplasty (RTSA) have been performed in complicated fracture cases, such as head split, head dislocation, and displaced 3-part and 4-part fractures. Furthermore, RTSA can provide more reliable clinical outcomes than hemiarthroplasty.^13^^,^^15^ Therefore, RTSA has been increasingly used for PHF over the last decade and is considered a viable alternative treatment.^3^^,^^44^ Some previous studies assesed the importance of tuberosity healing in RTSA and concluded that it remains controversial^7^^,^^20^; however, the clinical effectiveness of tuberosity healing has been recognized in terms of joint stability, long-term survival, and postoperative range of motion (ROM), especially in rotational movement.^4^^,^^8^^,^^9^^,^^18^^,^^20^^,^^23^^,^^31^ Various factors affect tuberosity healing, such as cement technique (including cement-induced thermal necrosis),^22^^,^^24^^,^^28^ bone graft,^28^ and implant characteristics (in growth or on growth).^25^^,^^26^ However, suture configuration to fix the greater tuberosity (GT) and lesser tuberosity (LT) remains crucial.^1^^,^^4^^,^^12^^,^^20^ Therefore, several surgical procedures have been reported to achieve robust fixation. The difficulty in tuberosity fixation is defined as follows: (1) Difficulty providing robust fixation to fragile bones in older adults and (2) The supraspinatus, infraspinatus, and teres minor muscles are attached to the GT, with the infraspinatus and teres minor acting as the external rotators. Furthermore, the subscapularis tendon, acting as an internal rotator, is attached to the LT. Therefore, the rotator cuffs attached to the GT and LT have opposing effects and find it difficult to apply compression force to the GT and LT fragments simultaneously. A previous study reported high tuberosity healing and low postoperative reduction loss rates using the turned stem tension band (TSTB) technique.^39^^,^^40^ This method is based on the tension band principle, and it can provide compression force to the GT and LT simultaneously by creating 2 hinges across the bone fragment, even for fragile fragments in older adults. When the supraspinatus muscle is resected, the transverse force vector mainly acts on the GT and LT fragments.^5^^,^^29^^,^^37^ Vertical sutures were added to reinforce the fixation in the original TSTB technique, although the supraspinatus tendon was resected.

The vertical sutures may be unnecessary in case the supraspinatus tendon was resected. Therefore, this study aimed to compare 2 methods, TSTB with or without vertical suturing for PHFs, and evaluate tuberosity healing and reduction loss rates. We hypothesized that the TSTB without vertical suture would provide tuberosity healing and reduction loss comparable to those with vertical sutures.

## Materials and methods

### Patient selection

This study was approved by our hospital’s institutional review board (No. 4337). Written informed consent was obtained from all patients at the time of surgical consent prior to enrollment. All eligible patients were successfully enrolled. This retrospective study included patients who underwent RTSA for complex PHFs performed by a single surgeon at a single institution between October 2014 and January 2021.

We performed RTSA for patients with acute PHFs, including those with (1) a dislocated acute 3-part and 4-part fracture,^30^ (2) 3-part and 4-part acute head-splitting fractures, (3) 3-part and 4-part acute anatomical neck fracture, and (4) age at the time of surgery >70 years.

Patients with old PHFs and revision cases (fracture sequelae, including failed conservative treatment) and paraplegia on the affected side due to a stroke or shoulder joint infection were excluded from the study. Overall, 41 patients underwent primary RTSA. Four patients in the vertical suture group were lost to follow-up. Two of these patients died because of reasons unrelated to the surgery. Finally, 35 patients who were followed up for >2 years were included in this study. The mean observation period was 31 (range, 24-60) months, and the mean patient age was 78.7 years (range, 70-87).

Two prostheses types were used for RTSA: (1) the trabecular metal reverse shoulder system (Zimmer Biomet, Warsaw, IN, USA), used for humeral stems and baseplates accompanied by angled-bony increased offset between October 2014 and 2018 (20 cases) and (2) the trabecular metal reverse shoulder system for humeral stems combined with the comprehensive shoulder system (Zimmer Biomet) (15 cases), used as the glenoid component between November 2018 and January 2021. Vertical suturing was discontinued in August 2018. The 35 patients were divided into 2 groups (vertical suture, 18 cases; without vertical suture, 17 cases), and the clinical outcomes and radiographic findings were assessed.

### Radiographic assessment and physical examination

The patients were evaluated at 2, 3, 4, 5, 6, 8, 10, 12, and 12 months thereafter. All clinical data were collected from medical records. Preoperative plain radiographs and computed tomography (CT) scans were obtained to grade PHF according to the Neer classification.^30^ Postoperative radiographs (anteroposterior and lateral views) were obtained at each follow-up visit. The reduction loss and bone absorption of the GT fragments were evaluated by comparing the initial postoperative and final follow-up radiographs on anteroposterior view. GT reduction was classified into 3 groups with respect to the upward migration of the GT fragment following the previous study: Group 1, fragment migration ≤2 mm; Group 2, fragment migration ≤5 mm; and Group 3, fragment migration ≥6 mm. Group 1 or 2 were considered as acceptable GT reduction.^40^ Tuberosity healing, defined by observed trabeculation between the stem and tuberosities, was evaluated using postoperative CT. In addition, tomosynthesis was performed in a coronal view (SONIALVISION G4; Shimadzu Corp., Kyoto, Japan). CT was performed in all patients at least 6 months postoperatively. Tomosynthesis is a technology that can obtain high-resolution tomographic images while irradiating them at certain angles by reducing metallic artifacts^17^^,^^19^^,^^42^^,^^43^ obtained at the final follow-up. Two surgeons not affiliated with the procedure performed the radiological evaluation (healing and reduction loss of the GT). Inter-rater reliability was evaluated using Cohen’s kappa statistic. In addition, bone resorption (presence or absence) associated with GT was recorded.

Active ROM (flexion, abduction, and external and internal rotation) was evaluated at the final follow-up. Except for internal rotation, ROM was evaluated using a goniometer. The vertebral level reached by the tip of the thumb was converted to a numerical score from the thigh (1 point) to the first thoracic vertebra (20 points) to evaluate internal rotation.^41^ All ROM data were evaluated by physiotherapists uninvolved in the surgery. The American Shoulder and Elbow Surgeons (ASES) and Numerical Rating Scale scores were evaluated at the final follow-up. Furthermore, other conditions, such as preoperative neurological disorders and complications associated with the surgery, were recorded.

### Surgical technique

All the surgeries were performed using the TSTB technique. The same stem was used for all patients (Trabecular Metal Reverse Shoulder System; Zimmer Biomet, Warsaw, IN, USA). The standard deltopectoral approach was used with the patient in the beach chair position under general anesthesia or a single interscalene block. Tenodesis of the long head of biceps tendon was routinely performed in the subpectoralis major position. The humeral head was carefully retrieved. One or 2 stay sutures (No. 2 Ethibond; Ethicon, Raritan, NJ, USA) were placed into the GT and LT bone tendon junction. Following a previous study reporting that the supraspinatus tendon resection contributes to a biomechanically stable tuberosity construct when performing RTSA for PHFs, we resected any remaining supraspinatus tendon.^5^^,^^29^^,^^37^ The glenoid surface was exposed after retracting the tuberosities, and the baseplate and glenosphere were implanted.

**Figure 1:**
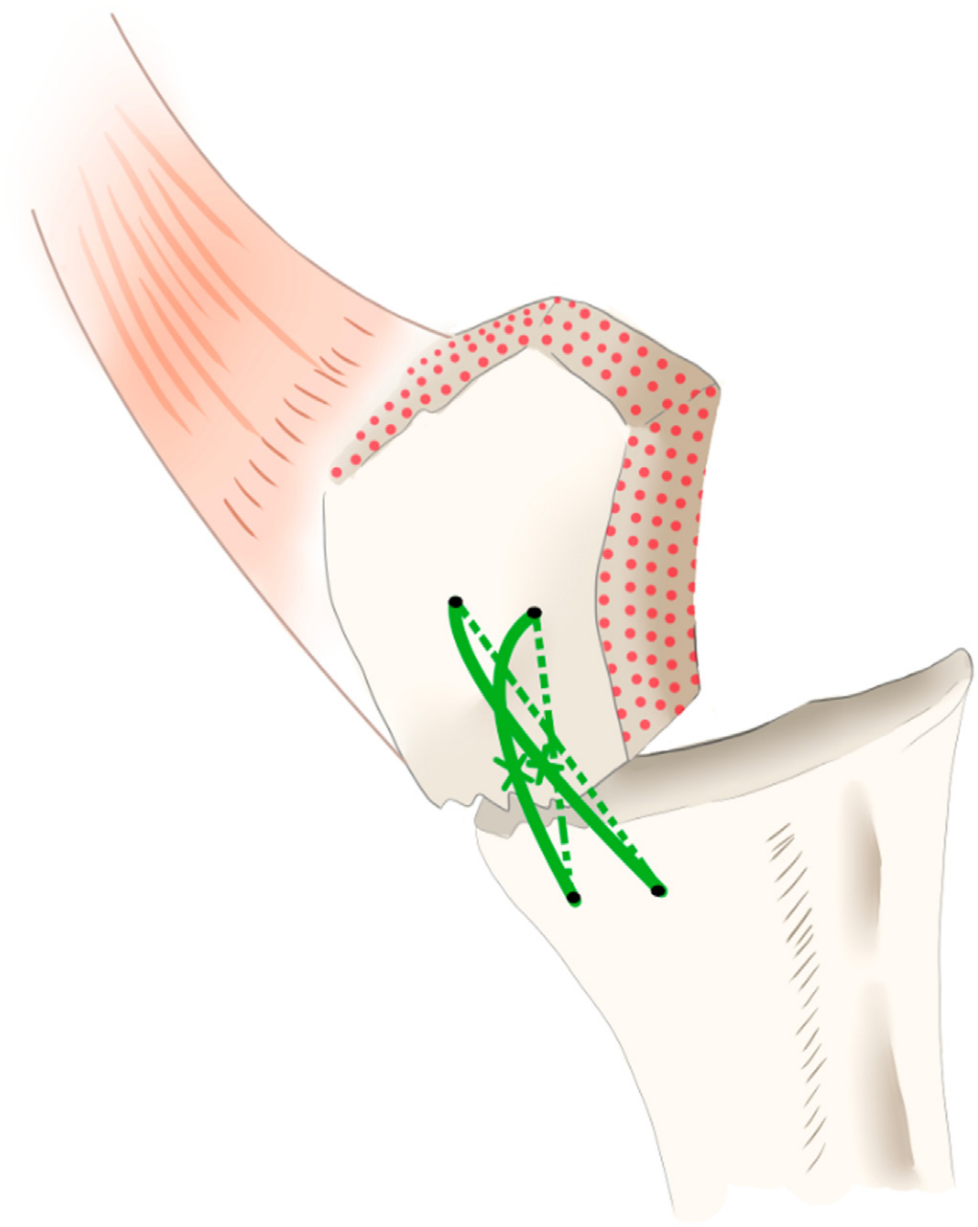
The humerus and greater tuberosity (GT) fragment were connected with vertical sutures. The drill holes were established at least 1 cm distal to the fracture site to avoid suture cutout. Modified from reference number.^39^

**Figure 2:**
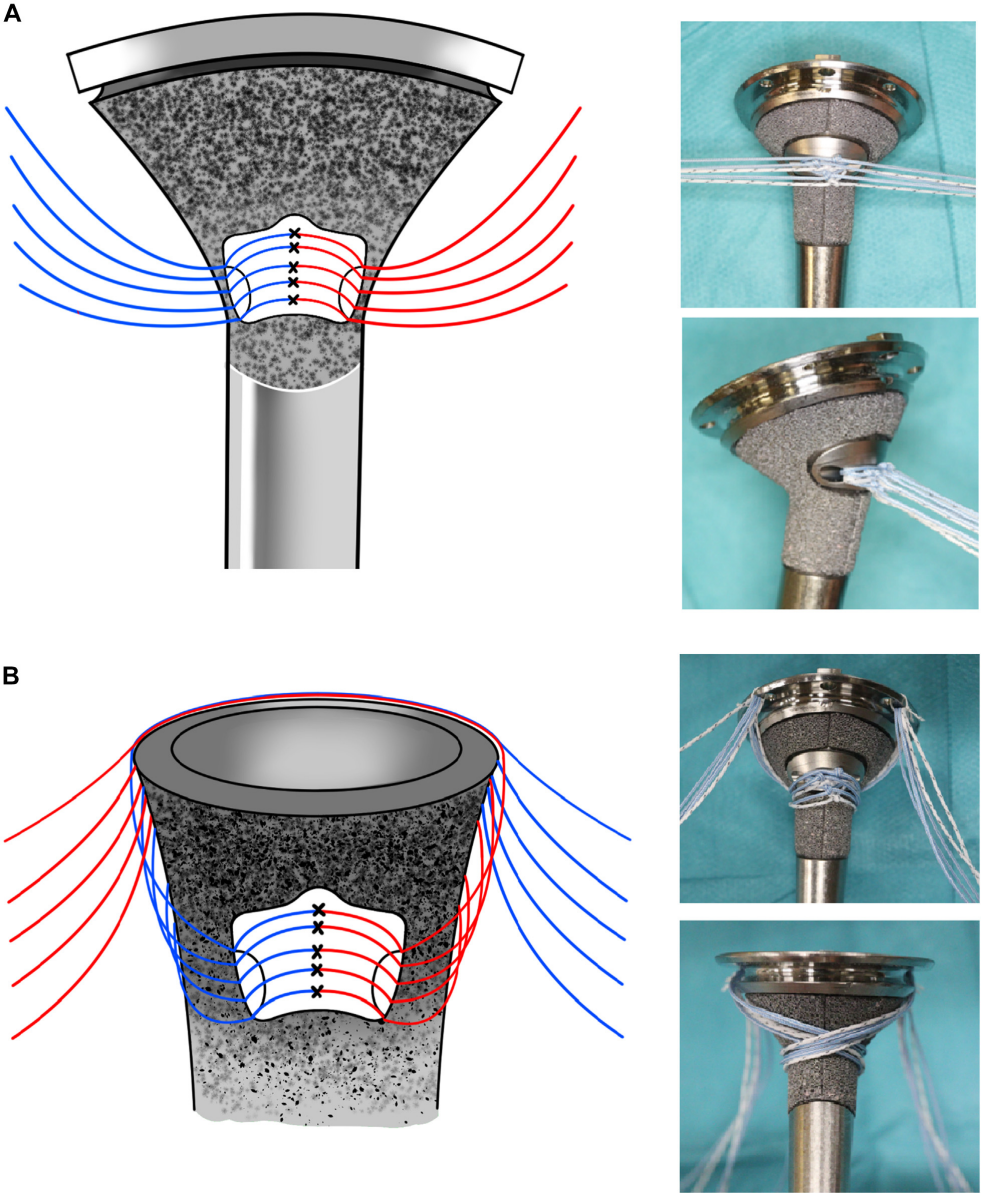
(**A**) At least 5 strong sutures were individually passed through the suture hole at the lateral side of the humeral stem and tied to avoid sliding. ×, suture knot. (**B**) Five sutures were turned counterclockwise, and the other 5 were turned clockwise around the stem. Modified from reference number.^39^

The definitive stem diameter was determined using the diameter of the last straight reamer. The stem height was determined based on the reduction in the GT fragment. In the vertical suture group, 1 or 2 nonabsorbable strong sutures (No. 2 FiberWire; Arthrex, Naples, FL, USA) were passed through holes drilled into the diaphysis of the humerus and GT fragments and secured as vertical sutures. Drill holes were created at least 1 cm from the fracture site to avoid suture cutout. The GT and LT are located across the bicipital groove. The bicipital groove was regarded as a useful landmark for anatomical reduction of GT and LT fragments. Approximately 1 cm lateral to the bicipital groove was used as a guide for the drill site (Fig. 1). Stem height was considered optimal when the stem summit was positioned at or slightly above the GT. Before inserting the stem into the humeral canal, at least 5 strong sutures were individually passed through the suture hole at the lateral side of the humeral stem and tied to avoid sliding (Fig. 2, *A*); therefore, each suture pair did not require being tied, and confusion was less likely to occur with the increasing number of sutures. In addition, it helps to make a “hinge point.” Five sutures were turned counterclockwise, and the other 5 were turned clockwise around the stem (Fig. 2, *B*). To avoid thermal bone necrosis induced by the cement,^11^^,^^22^^,^^28^ any cement 2 cm distal to the fracture line was removed.^22^ All humeral stems were cemented using a cement gun and positioned at 20° of retroversion.

**Figure 3:**
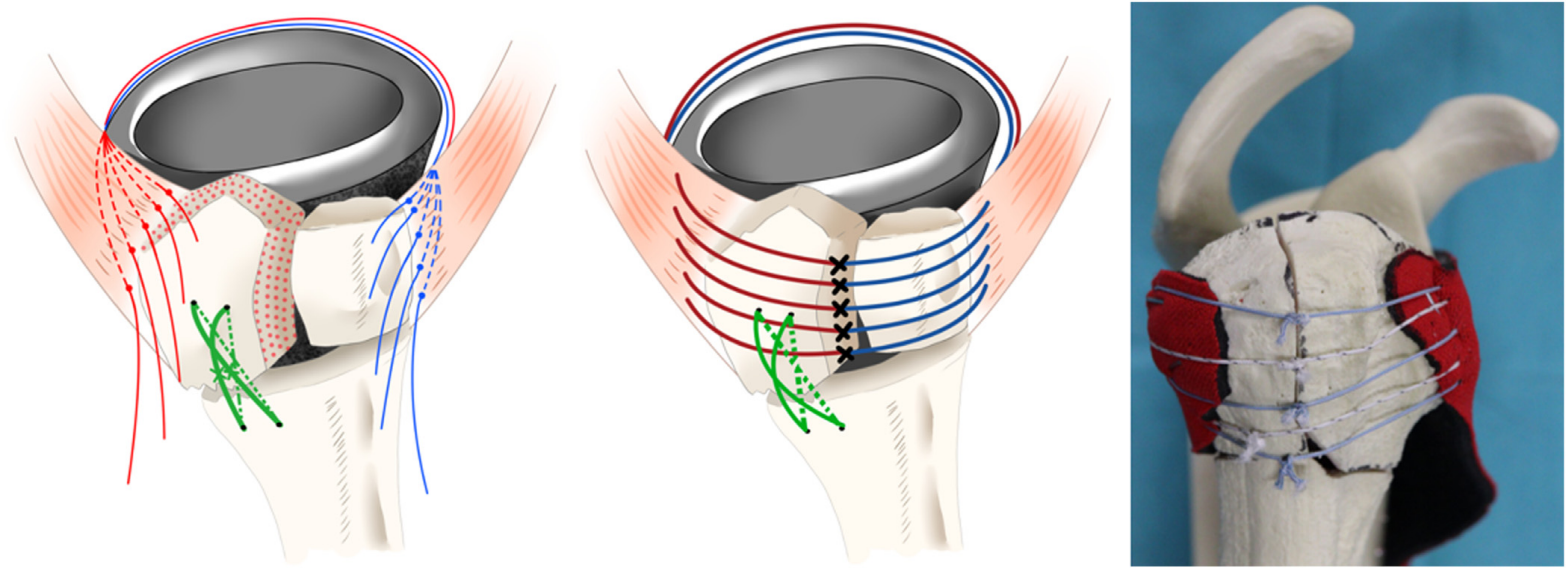
Five sutures were turned counterclockwise around the stem; 2 were applied to the teres minor muscle and 3 to the infraspinatus tendon. The other 5 sutures turned clockwise around the stem were applied to the subscapularis tendon. All sutures were passed through the bone tendon junction and were tied tightly. Modified from reference number.^39^

Of the 5 sutures turned counterclockwise around the stem, 2 were applied to the teres minor muscle and 3 to the infraspinatus tendon. The other 5 sutures turned clockwise around the stem were applied to the subscapularis tendon. All sutures were passed through the bone-tendon junction. After inserting the polyethylene spacer, the stem and glenosphere were repositioned, and the cancellous bone harvested from the fractured humeral head was packed into the gap between the tuberosity and stem to avoid an initial gap. The sutures were tied tightly (Fig. 3). Internal and external rotations were performed to confirm adequate fixation of the GT and LT fragments, and the surgery was completed.

### Postoperative protocol

All patients wore a shoulder abduction sling at 30° for 5 weeks. Active hand and elbow exercises were encouraged starting from 1 day postoperatively. The shoulder exercises were performed under the supervision of a physical therapist while wearing an abduction sling. Passive and active shoulder elevation exercises in the supine position were initiated at postoperative 2 and 5 weeks, respectively, and active exercises in the sitting position began 6 weeks postoperatively. Rotational exercises were initiated 4 weeks postoperatively.

### Statistical analysis

Continuous variables (ROM and age) and categorical variables (sex and dominant hand involvement) were compared using the Mann–Whitney U and Fisher’s exact tests, respectively. The Wilcoxon signed-rank test was used to compare the preoperative and postoperative ROM and ASES scores. Inter-rater reliability was evaluated using Cohen’s kappa statistics. Statistical significance was set at *P* < .05. All statistical analyses were performed using the EZR software (Saitama Medical Center, Jichi Medical University, Saitama, Japan), a modified version of R commander (The R Foundation for Statistical Computing, Vienna, Austria).

## Results

### Baseline characteristics of the study participants

The baseline characteristics of the study participants are summarized in Table I. The study participants showed no significant differences in age, dominant side, rotator sex, and fracture type. Nine of the 18 (50%) patients with 4-part dislocated fractures had neurological disorders from the injury time. The neurological disorder improved in 8 patients; however, no improvement was observed in the remaining 1 patient.

**Table I:** Baseline characteristics of with and without vertical suture groups.

	Vertical suture group (n = 18)	Without vertical suture group (n = 17)	*P* value
Age, y	80.4 ± 4.7	76.9 ± 5.8	.084
Dominant side	11 (61%)	11 (65%)	1.000
Male	3 (16.6%)	8 (47.0%)	.075
Type of fracture	3-part anatomical neck: 2	3-part anatomical neck: 1	.536
	4-part anatomical neck: 2	4-part anatomical neck: 2	
	3-part head splitting: 1	3-part head splitting: 0	
	4-part head splitting: 2	4-part head splitting: 1	
	3-part dislocation: 1	3-part dislocation: 5	
	4-part dislocation: 10	4-part dislocation: 8	
Preoperative neurological disorders	Median, axillary nerve: 1	Median, radial, ulnar nerve: 1	
	Musculocutaneous, axillary nerve: 2	Musculocutaneous, axillary nerve: 1	
	Radial, axillary nerve: 1	Axillary nerve: 2	
	Axillary nerve: 2		
Follow-up period, mo	34.3 (range 24-60)	29.1 (range 24-40)	

Continuous variables are presented as a mean ± standard deviation.

### Comparison of radiographic analyses, ROMs, and ASES scores

**Figure 4:**
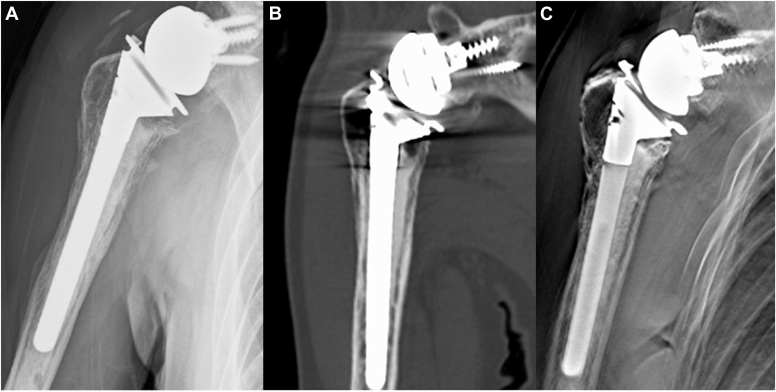
(**A**) A plain radiograph. No reduction loss was observed. (**B**) A computed tomography (CT). Metallic artifacts were observed to make evaluation difficult. (**C**) Tomosynthesis can obtain high-resolution tomographic images by reducing metallic artifacts.

Table II shows the radiographic analyses, ROMs, and ASES scores. No significant differences were observed in reduction loss rates, bone absorption, tuberosity healing, Numerical Rating Scale scores, ROMs, or ASES scores. No patient was categorized into Group 3, and all patients were considered to achieve acceptable GT reduction in both groups. The bone healing rate was 100% in both groups, consistent with the tomosynthesis results (Fig. 4, *A*-*C*). The Cohen’s kappa coefficient for radiologic assessment was 1.0.

**Table II:** Comparison with and without vertical suture groups.

	Vertical suture group (n = 18)	Without vertical suture group (n = 17)	*P* value
Reduction loss	Group 1:14 cases	Group 1:16 cases	.34
	Group 2:4 cases	Group 2:1 case	
	Group 3:0 case	Group 3:0 case	
Bone absorption	4/18 cases, 22.2%	6/17 cases, 35.3%	.47
Bone healing rate	100%	100%	1.0
Numerical rating scale	1.2 ± 0.9	0.8 ± 0.9	.13
Flexion (°)	119 ± 33	124 ± 23	.95
Abduction (°)	116 ± 35	115 ± 27	.78
External rotation (°)	27 ± 12	21 ± 8	.16
Internal rotation	6 ± 4 (L3)	6 ± 2 (L3)	.87
ASES score	77.3 ± 10.7	81.6 ± 6.3	.59

*ASES*, American Shoulder and Elbow Surgeons; *L*, lumbar level.

Continuous variables are presented as a mean ± standard deviation.

Patients with shattered GT fragments tended to have bone absorption.

## Discussion

The results of this study support our hypothesis that TSTB without vertical sutures provided tuberosity healing and reduction loss comparable to that of TSTB with vertical sutures. Tuberosity healing rates were 100% in both groups, suggesting that the TSTB achieves robust fixation based on the tension band principle regardless of the vertical sutures.

**Figure 5:**
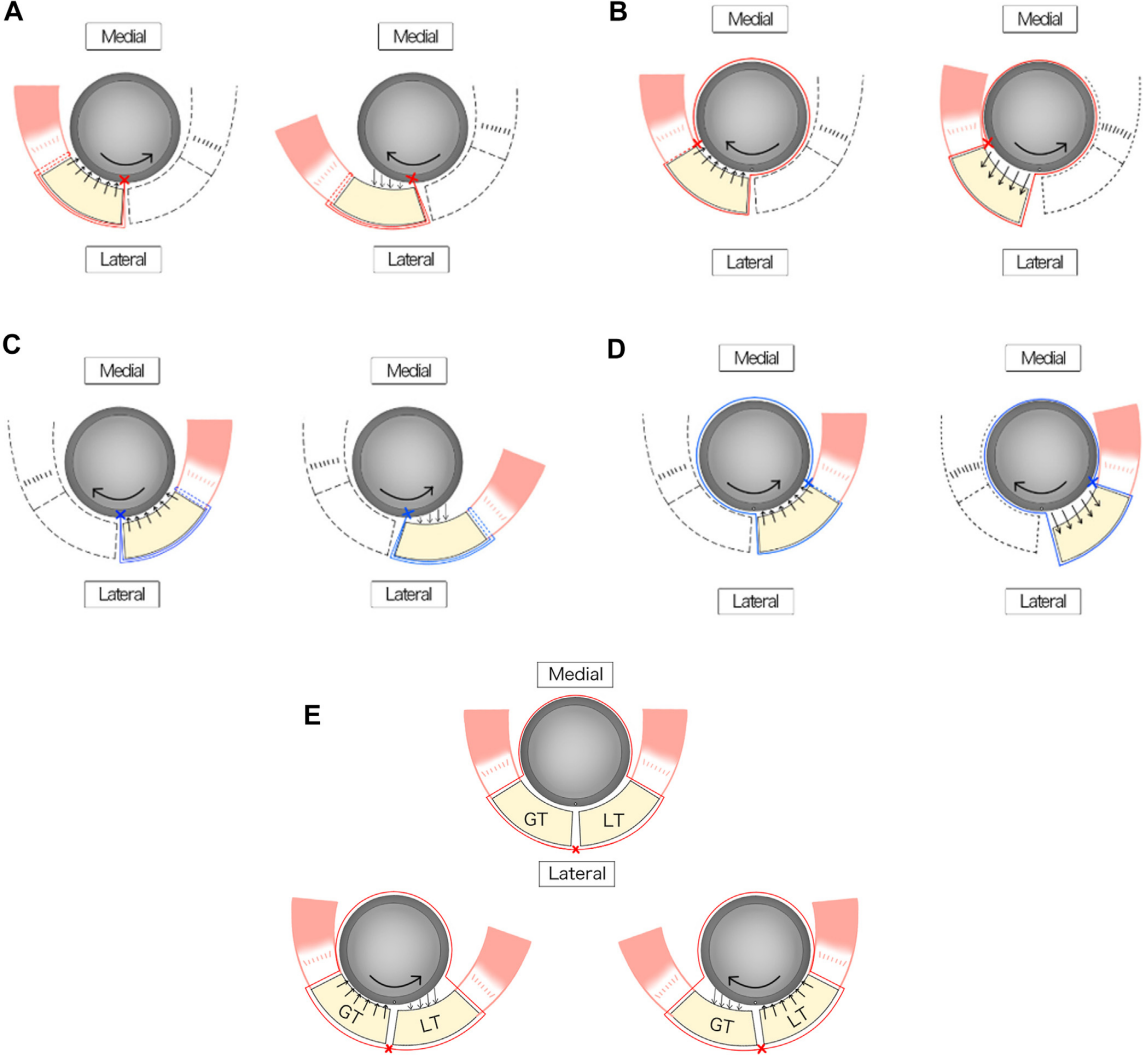
(**A)** The hinge is placed on the lateral side of the greater tuberosity (GT) fragment using the suture hole. In internal rotation, compressive force is applied to the GT fragment, and tensile force is applied to the GT fragment in external rotation. (**B**) The hinge was placed on the medial side of the GT fragment. A compressive force was applied to the GT fragment during external rotation, and a tensile force was applied during internal rotation. (**C**) The hinge was placed on the lateral side of the lesser tuberosity (LT) fragment using a suture hole. A compressive force was applied during external rotation, whereas a tensile force was applied during internal rotation. (**D**) The hinge is placed on the medial side of the LT fragment. During internal rotation, a compressive force was applied to the LT fragment. During external rotation, a tensile force was applied to the LT fragment. (**E**) The cerclage wiring method is shown, in which only the lateral hinge is formed. During internal rotation, a compressive force was applied to the GT, whereas a tensile force was applied to the LT. During external rotation, a tensile force was applied to the GT, whereas a compressive force was applied to the LT. Modified from reference number.^39^

**Figure 6:**
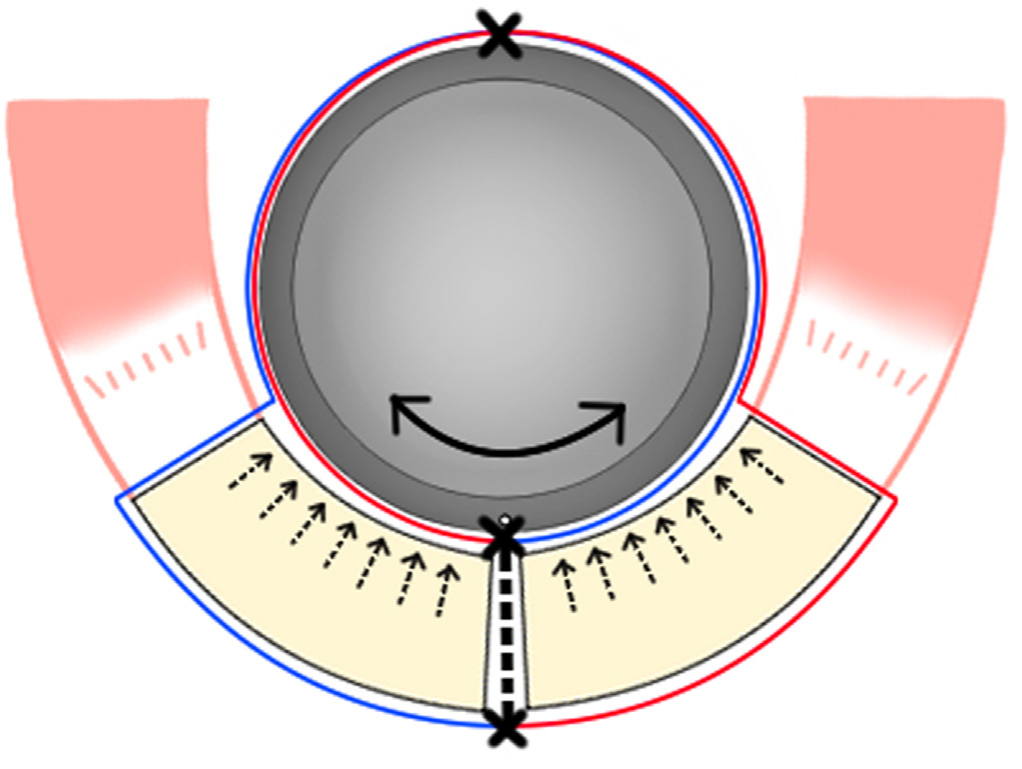
The turned stem tension band technique (TSTB) created 2 hinges by turning the sutures around the stem in clockwise and counterclockwise directions and passing them through the greater tuberosity (GT) and lesser tuberosity (LT) fragments and applying a compressive force to the GT and LT fragments simultaneously regardless of rotational movements. ×, hinge point. Modified from reference number.^39^

Tuberosity healing has several advantages in implant survival, joint stability,^21^^,^^34^ avoidance of infection,^13^^,^^18^^,^^20^^,^^31^ and rotational ROMs.^4^^,^^18^^,^^20^^,^^31^ The compression force between the fragments (infrafragmentary compression) and mechanical stability is essential for fracture healing. The infraspinatus and teres minor (external rotators) tendons stop at the GT. In contrast, the subscapularis (internal rotator) tendon stops at the LT. Consequently, bone healing of the GT and LT requires a compression force and mechanical stability for bone fragments to which muscles with opposite functions are attached. In addition to these mechanical problems, achieving a robust fixation for fragile bone in older adults can be difficult. Previous studies have reported surgical techniques for tuberosity repair with tuberosity healing rates ranging from 47% to 100%,^4^^,^^6^^,^^15^^,^^23^^,^^27^ and nonunion frequently occurs in older women because of poor bone quality.^14^ Notably, most of these previously reported surgical techniques are considered cerclage wiring methods.^1^^,^^2^^,^^4^^,^^12^^,^^36^ This method achieves fixation strength through the frictional force between the sutures (wires) and the bone fragments and, therefore, does not apply compression force to the GT and LT regardless of rotational movement (Fig. 5, *E*). However, the TSTB can apply a compression force to the GT and LT, regardless of the rotational motion, based on the tension band principle. The tension band is a technique in which the tension applied to bone fragments is neutralized by creating a hinge and converted to a compression force, resulting in dynamic stability. Therefore, creating 2 hinges across the bone fragment is important to simultaneously applying a compressive force to the GT and LT fragments regardless of the rotational movement (Fig. 5, *A*-*D*). TSTB creates 2 hinges by turning the sutures around the stem in the clockwise and counterclockwise directions and passing them through the GT and LT fragments (Fig. 6). In our study, the mean age of the patients was 78.7 years (range, 70-87), and the proportion of female patients was 68.5% (24/35 cases), suggesting that they may have poor bone quality. However, the bone healing rate was 100%, indicating that TSTB can provide robust fixation regardless of bone quality.

**Figure 7:**
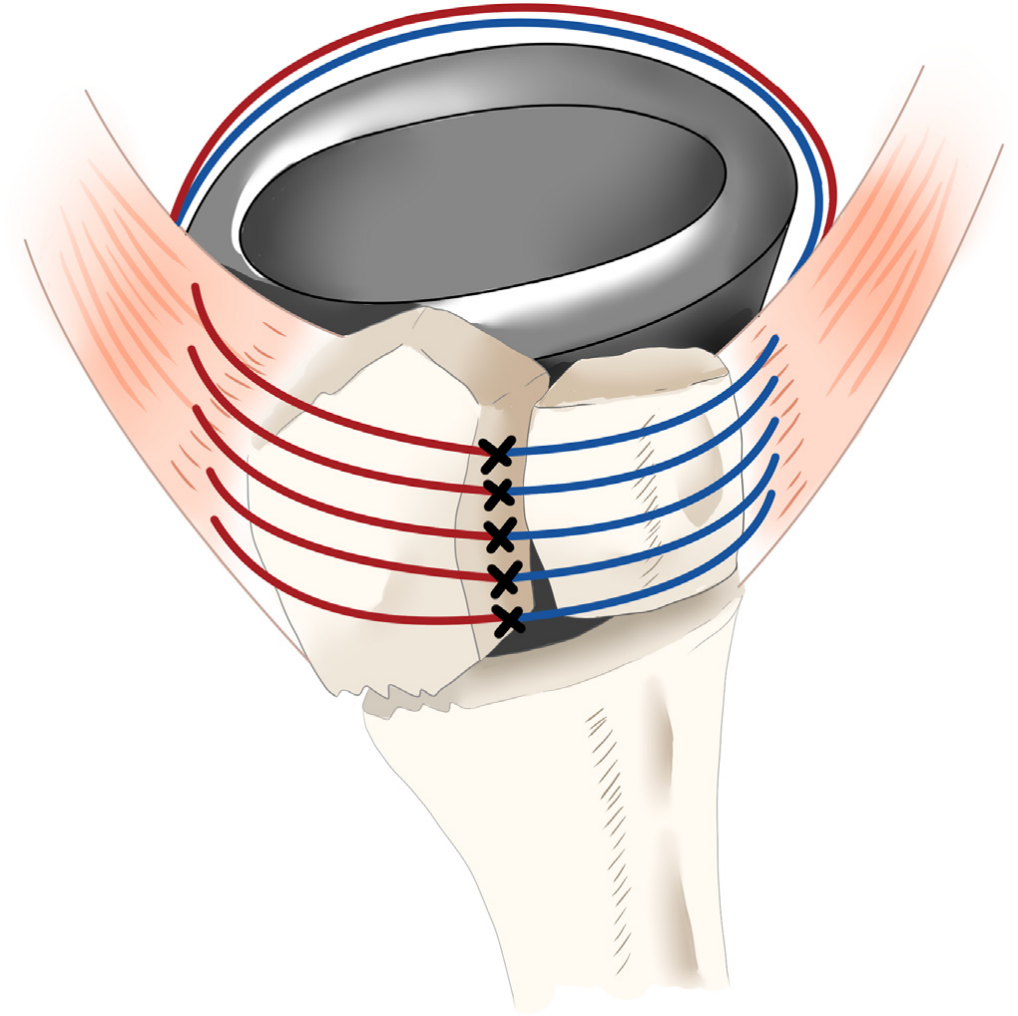
Turned stem tension band technique (TSTB) without vertical suture.

The results of our study demonstrated that TSTB without vertical sutures achieved a high rate of bone healing (100%) and a low rate of reduction loss comparable to that of TSTB with vertical sutures. The rotator cuffs act as antagonist in RTSA.^16^ We resected the supraspinatus muscle to prevent reduction loss and lower the GT fragment in this series.^5^^,^^29^^,^^37^ Resection of the supraspinatus muscle facilitates the repositioning of the GT to its anatomic position,^5^^,^^29^^,^^37^ and consequently, only the muscles involved in rotational movements are attached to the GT and LT. In other words, only the force vectors in the transverse direction were in effect. In addition, the LT and GT were attached to the stem through the suture hole using the TSTB technique; therefore, vertical sutures may be unnecessary (Fig. 7).

CT was used to detect bone in-growth, bone incorporation, and radiolucent zones. However, a previous study reported that CT could not accurately detect bone graft resorption (sensitivity: 38%, accuracy: 46%). The bone resorption gap was consistently underestimated,^10^ suggesting that CT cannot detect bone healing because of metal artifacts. Tomosynthesis detected radiolucent lines around metallic implants more accurately than CT and plain radiography, and it improved the diagnostic accuracy of detecting signs of bone incorporation (spot welds).^19^^,^^32^^,^^33^^,^^38^^,^^42^^,^^43^ The results of this study could not demonstrate the clinical superiority of tomosynthesis because the radiologic evaluations using CT and tomosynthesis were in perfect agreement. However, given the reduced metal artifacts and radiation exposure compared with that via CT, tomosynthesis may be used as a standard modality in the future to evaluate bone healing.^32^^,^^33^^,^^38^

Patients with shattered GT fragments tended to have bone absorption. However, none of the cases had complete resorption. In such cases, it can be difficult to evaluate bone healing. Tomosynthesis can detect spot welds more accurately than CT and plain radiography.^19^^,^^38^^,^^42^ Tomosynthesis has allowed us to more accurately assess bone healing, even in patients with bone resorption. The bone resorption has been caused by impaired blood flow due to the severe comminution of bone fragments. Alternatively, reducing the number of sutures or using tape materials may be necessary since a blood flow obstruction due to several sutures is possible.

This study has some limitations. First, the number of patients was small, and a power analysis was lacking. There were no previous studies comparing GT reduction methods, and therefore no priori analysis could be performed. A post hoc analysis was performed for the comparison of the 2 groups, but it was underpowered. This is because the difference between the 2 groups was so small that a large number of cases would be required to achieve adequate power. Second, the LT fragment reduction loss was not evaluated because it was difficult to precisely assess the LT position on radiographs. CT was not performed immediately postoperatively; thus, we could not compare CT obtained immediately postoperatively and after tuberosity healing. The tomosynthesis used in this study was coronal view only; therefore, the LT was not evaluated.

Regardless, this study demonstrates that TSTB can achieve high tuberosity healing and low reduction loss rates with its simple suture configuration, which is clinically relevant.

## Conclusion

The TSTB technique for PHFs provided high tuberosity healing and low reduction loss rates regardless of the vertical sutures. This technique is easily adoptable and reproducible; therefore, it may be an effective treatment for PHFs in older adults.

## Disclaimers:

Funding: No funding was disclosed by the authors.

Conflicts of interest: The authors, their immediate family, and any research foundation with which they are affiliated did not receive any financial payments or other benefits from any commercial entity related to the subject of this article.
